# Insights into the control of metabolism and biomass accumulation in a staple C_4_ grass

**DOI:** 10.1093/jxb/eraa307

**Published:** 2020-09-19

**Authors:** Kumari Billakurthi, Tina B Schreier

**Affiliations:** University of Cambridge, Department of Plant Sciences, Downing Site, Cambridge, UK

**Keywords:** Biomass and yield, DAHPS development, metabolism, shikimate pathway

## Abstract

This article comments on:

**Chen J, Zhu M, Liu R, Zhang M, Lv Y, Liu Y, Xiao X, Yuan J, Cai H**. 2020. BIOMASS YIELD 1 regulates Sorghum biomass and grain yield via the shikimate pathway. Journal of Experimental Botany **71**, 5506–5520.

**The shikimate pathway is key to the synthesis of aromatic amino acids and many secondary metabolites in plants.** Chen *et al.* (2020) **identified the *biomass yield 1* (*by1*) mutant in Sorghum using an ethylmethane sulfonate (EMS) mutagenesis screen. The mutation mapped to an amino acid substitution in the sequence of** **d** **-arabino-heptulosonate-7-phosphate synthase (DAHPS)—the enzyme catalysing the first committed step of the shikimate pathway. Reduced DAHPS activity in *by1* mutants resulted in severe defects in growth and development, such as reduced height, narrow leaves and stem, and abnormal floral organs, which ultimately led to less biomass and yield production. The *by1* mutant reveals novel information regarding the integration of primary and secondary metabolism and how this influences growth and development.**

The shikimate pathway in chloroplasts is an important link between primary and secondary plant metabolism. It is composed of seven steps, starting with the substrates phospho*enol*pyruvate (PEP) and erythrose-4-phosphate (E4P), produced by the glycolytic and pentose phosphate pathways, respectively. The pathway results in the formation of chorismate, which serves as a precursor molecule for the synthesis of the aromatic amino acids (AAAs); Phe, Tyr, and Trp (Box 1). In plants, AAAs are not only essential building blocks for protein synthesis, but are also precursors for many secondary metabolites including flavonoids, lignin, and hormones (auxin and salicylic acid) (Maeda and Dudareva, 2012).

**Box 1. The shikimate pathway in plants**

The shikimate pathway converts the substrates phospho*enol* pyruvate (PEP) and erythrose-4-phosphate (E4P) into chorismate in seven enzymatic steps. Chorismate is the precursor for aromatic amino acids and many secondary metabolites. The *by1* mutation in Sorghum was mapped to an amino acid substitution in the enzyme catalysing the first committed step in the shikimate pathway, the DAHP synthase (highlighted in red).

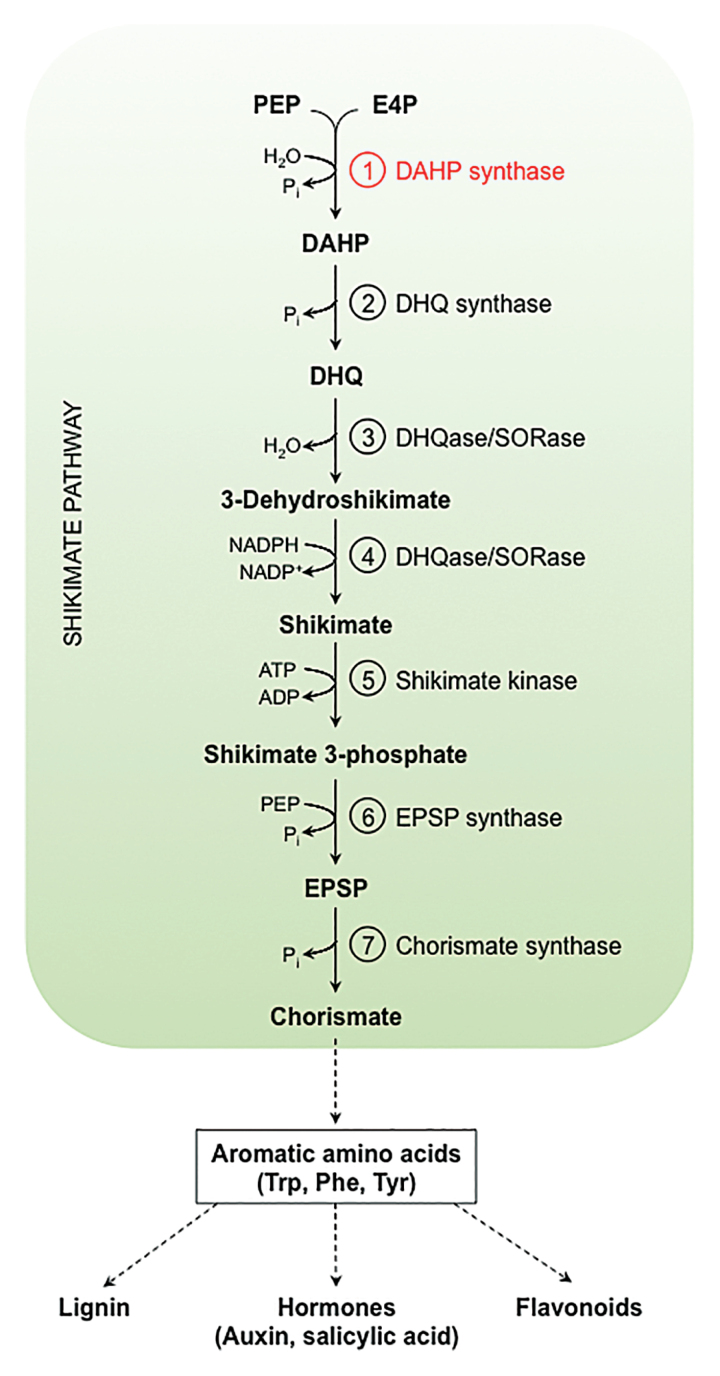

Abbreviations: Phospho*enol*pyruvate (PEP), erythrose-4-phosphate (E4P), 3-deoxy-d-*arabino*-heptulosonate-7-phosphate (DAHP), 3-dehydroquinate (DHQ), 3-dehydroquinate dehydratase/shikimate:NADP oxidoreductase (DHQase/SORase), 5-*enol*pyruvylshikimate-3-phosphate (EPSP).

The shikimate pathway is required for growth and development. The well-known herbicide, glyphosate, inhibits 5-enolpyruvylshikimate-3-phosphate (EPSP) synthase—an enzyme involved in the shikimate pathway. Also, mutations of various shikimate enzymes were identified in the screen for embryo-lethal mutants in Arabidopsis (Tzafrir *et al.*, 2004; Pagnussat *et al.*, 2005). Despite its importance, it is still not completely understood how the shikimate pathway in plants is regulated. Although the existence of feedback regulation by the levels of AAAs and metabolites derived from them has been shown in a few previous studies, the molecular mechanism remains elusive (Maeda and Dudareva, 2012).

## Sorghum *BIOMASS YIELD 1* (*BY1*) encodes DAHPS and regulates plant metabolism and development

As Sorghum is an important staple food and biofuel crop, Chen *et al.* (2020) performed an EMS mutagenesis screen in *Sorghum bicolor* to isolate candidate genes regulating biomass production and grain yield. The mutant *by1* showed a drastic reduction in both biomass and yield. The characteristic growth features associated with decreased biomass of *by1* were reduced plant height, thinner stems, and smaller, narrower leaves. In *by1*, internode, leaf blade, and vascular cells were developed normally except that the final cell volume was significantly reduced. The authors conclude that reduced cell expansion was associated with abnormal growth phenotypes of *by1*, which ultimately resulted in decreased plant biomass. Developmental defects including poorly developed panicles, anthers, and pollen contributed to the final reduction of grain yield in *by1*. In *by1*, anthers were thin and small and, moreover, pollen viability was reduced by ~50% and pollen grains appeared shrunken.

To map the causative gene responsible for the *by1* phenotype, *by1* was outcrossed to Shangzhuang broomcorn. Using a map-based cloning approach, an EMS-induced single single nucleotide polymorphism (SNP; cytosine to thymine) was mapped to the Sobic.002G379600 (*BY1*) gene, which resulted in a Pro to Leu amino acid substitution (Pro192Leu) in the protein product. The gene *BY1* encodes a DAHPS, which catalyses the first reaction in the shikimate pathway. The authors report that the Pro192Leu substitution reduced the BY1 enzyme activity by ~33%. *BY1* function was validated in rice by targeting Loc_Os07g42960 (a homologue of Sorghum *BY1*) by CRISPR/Cas9 [clustered regularly interspaced palindromic repeats (CRISPR)/CRISPR-associated protein]. Growth and developmental abnormalities were similar to Sorghum *by1*, and both biomass and yield were also drastically decreased.

Chen *et al.* (2020) analysed the leaf transcriptome and metabolome of the wild type and *by1* to dissect the developmental pathways that were affected in the mutant line. Most of the genes involved in pathways operating upstream of the shikimate pathway (photosynthesis, glycolysis, and the pentose phosphate pathway) and the phenylpropanoid pathway that operates downstream of the shikimate pathway were significantly up-regulated. In contrast, the levels of metabolites in those pathways including PEP, shikimate, Phe, and chalcone were reduced. Interestingly, most of the metabolites with significantly lowered levels were phenylpropanoids, and 19 of 22 flavonoids were less abundant. The authors propose that a positive feedback signal in response to reduced metabolites enhances the expression of genes involved in the shikimate pathway and its upstream pathways to restore the carbon flux into the shikimate pathway. This is in agreement with previous studies that showed that shikimate pathway genes in response to AAAs and metabolite levels were regulated at the gene expression level rather than at the post-translational level (Maeda and Dudareva, 2012).

The reported developmental defects in the *by1* mutant are interesting given that previous works on DAHPS orthologues have also mainly proposed roles in secondary metabolism. In Arabidopsis and cotton, DAHPS expression is induced upon wounding or pathogen infection (Keith *et al.*, 1991; Yang *et al.*, 2015). RNAi silencing of *PhDAHP1* in Petunia, one of the two DAHPS isoforms, leads to a reduction of floral volatile benzoid/phenylpropanoids levels (Langer *et al.*, 2014).

## Future perspectives

This work is interesting in the context that Sorghum is an important C_4_ grass belonging to the NADP-ME subtype. In C_4_ plants, PEP is the main carbon acceptor of the initial CO_2_ fixation via PEP carboxylase leading to the formation of the four-carbon acid. As the metabolite profiling in the *by1* mutant showed low PEP levels, it would be interesting to know how this impacts the carbon flux through the C_4_ pathway and ultimately photosynthetic efficiency. Low photosynthetic efficiency may underpin the reduction in biomass and grain yield in the *by1* mutant.

Plant growth in *by1* could also be affected due to reduced cell expansion. Levels of AAAs including Trp and most of the flavonoids were lowered in *by1*. The plant hormone auxin, which is a major regulator of plant growth and development, exerts many of the developmental processes through cell division and expansion (Perrot-Rechenmann, 2010), and polar auxin transport is known to control these processes (Michniewicz *et al.*, 2007). Auxin is mainly synthesized from the amino acid Trp (Trp-dependent pathway); however, it can also be produced via a Trp-independent pathway. The branch point of both pathways, indole-3-glycerol phosphate, is an intermediate product of the Trp biosynthesis pathway (Ouyang *et al.*, 2000). The Arabidopsis, *indole-3-glycerol phosphate synthase* (*IGS*) and *tryptophan synthase* mutants were smaller and free auxin levels were reduced in *IGS* mutants (Last *et al.*, 1991; Ouyang *et al.*, 2000). Interestingly, it has been shown that flavonoids function as endogenous regulators of polar auxin transport. In the flavonoid-deficient Arabidopsis mutant *transparent testa4* (*tt4*), plant height and inflorescence stem thickness were decreased due to elevated basipetal auxin transport (Brown *et al.*, 2001). Furthermore, the root gravitropic response was delayed in *tt4* due to failure in the prompt establishment of the auxin gradient (Buer and Munday, 2004). Some of the auxin efflux transporters (PINs) were mislocalized in flavonoid-deficient mutants (Peer *et al.*, 2004). However, the level of conservation of the mechanism of flavonoids as negative regulators of auxin transport is unknown. Therefore, measuring free auxin levels and studying auxin transport in the *by1* mutant might help to dissect the contribution of auxin to the developmental defects observed in *by1*.

In *by1*, 50% of the pollen grains were sterile due to a poorly developed pollen wall. What possible mechanisms could be involved? It has been shown that pollen integrity is associated with its viability (Zhang *et al.*, 2007; Ren *et al.*, 2020). The pollen wall is developed in a stepwise process: the microspore mother cell is surrounded by a temporary callose wall, and its timely degradation is important for the release of newly formed microspores from tetrads and formation of exine (outer pollen wall). Pollen wall development and cell integrity were impaired in Arabidopsis *ms188* (*MYB103*) and rice *dmd1* (*defective microspore development1*) knockout mutants that showed a delay in the timely degradation of the callose wall (Zhang *et al.*, 2007; Ren *et al.*, 2020). This ultimately resulted in male sterility. Moreover, flavonoids are known to be involved in pollen development and viability, and their role as reactive oxygen species (ROS) scavengers is crucial in maintaining pollen viability under heat stress conditions (Santiago and Sharkey, 2019). Therefore, in-depth cytological and transcriptome/proteome/metabolome analyses of anthers and/or pollen would be helpful to understand the developmental and molecular mechanisms behind abnormal anthers and pollen of *by1*. This would have broader applications in breeding male-sterile hybrids for important agronomic traits.

The amino acid substitution Pro192Leu could affect DAHPS activity in many different ways. The authors propose that the *by1* mutation changes the tertiary structure of the protein and this causes a reduction in enzyme activity. Previous work on the Arabidopsis DAHPS enzyme activity showed that DAHPS is subjected to redox regulation via the thioredoxin (TRX)/ferredoxin (Fd) system (Entus *et al.*, 2002). Further research would be needed to validate the change in tertiary structure, and its effect on substrate binding and redox regulation to work out precisely how the mutation compromises enzyme activity.

In summary, Chen *et al.* (2020) report that reduced activity of DAPHS alters the homeostasis between primary and secondary metabolism, causing growth and developmental abnormalities in Sorghum. The transcriptome and metabolite data sets from this study will be useful in future to increase our understanding of the shikimate pathway and its link to primary and secondary metabolism in Sorghum.
